# A novel fluorimetric method for determination of pseudoephedrine hydrochloride in pharmaceutical formulations and blood serum

**DOI:** 10.3906/kim-1912-6

**Published:** 2020-06-01

**Authors:** Nazli FARAJZADEH, Nasrin RANJBAR NADER

**Affiliations:** 1 Department of Chemistry, Faculty of Science and Technology, İstanbul Technical University,İstanbul Turkey; 2 Department of Analytical Chemistry, Faculty of Chemistry, University of Tabriz, Tabriz Iran

**Keywords:** Pseudoephedrine hydrochloride, gold nanoparticles, serum, pharmaceutical formulations, fluorescence

## Abstract

An inexpensive, simple, highly sensitive, and rapid fluorimetric method was developed for the analysis of pseudoephedrine hydrochloride at trace levels. The method is based on the recovery of fluorescence of Rh6G dye due to the interaction of pseudoephedrine hydrochloride with Rh6G-Au NPs complex, which results in the release of Rh6G from the complex and halting fluorescence resonance energy transfer between Rh6G and Au NPs. The intensity of fluorescence was directly proportional to the concentration of the analyte, which was used for its determination. Experimental factors were optimized by response-surface methodology. Under optimum conditions, the calibration curve was linear over the range of 15–150 ng mL^-1^ and the limit of detection (LOD) was 10 ng mL^-1^. Percent relative standard deviation (n= 5) for determination of 50 ng mL^-1^ pseudoephedrine hydrochloride was 3.74%. The method was successfully used for determining the analyte in human blood serum and in pharmaceutical formulations. The possible mechanistic description of the analytical reaction was proposed on the basis of TEM, FT-IR, and fluorescence spectra analysis.

## 1. Introduction

Pseudoephedrine hydrochloride (PSH), (1 *S* ,2 *S* ) -2-(methylamino)-1-phenylpropan-1-ol hydrochloride [1] is an orally active direct sympathomimetic phenethylamine that mainly works on the basis of its indirect influence on the adrenergic receptor system via displacement of norepinephrine from storage vesicles in presynaptic neurons. PSH also has nasal and bronchial decongestant activity, i.e. it increases the drainage of sinus secretions and opens blocked Eustachian tubes, hence it is used for medical treatment of the common cold and bronchitis [2–5]. This same effect can also cause hypertension, which is regarded as a side effect of PSH. Its other side effects are tachycardia and stimulation of the central nervous system [1,3,4]. In addition to the common cold and bronchitis, PSH is widely used singly or in combination with other drugs for the clinical treatment of seasonal allergic rhinitis, hay fever, and upper respiratory allergies [5]. A survey of the literature shows that various analytical methodologies have been used for determination of PSH, including gas chromatography (GC) [6], high-performance liquid chromatography (HPLC) [1,7], capillary electrophoresis (CE) [8,9], spectrophotometry (either as a method of analysis or as a detection system for methods using HPLC) [10–12], near-IR spectroscopy [13], radioimmunoassay [14], and voltammetry [15]. Most of these methods are expensive, time-consuming, and use multiple techniques or need tedious and labor-intensive sample pretreatment prior to analysis. Although some HPLC- [16] and CE-based [9] methods of analysis use laser-induced fluorescence detection, to the best of our knowledge, no more than one fluorescence analysis method has been proposed for determination of PSH [17]. In the present paper, we report a very sensitive, simple, fast, and inexpensive method for the determination of PSH in pharmaceutical formulations and serum samples. The method is based on the interaction of PSH with rhodamine 6G-treated gold nanoparticles (Rh6G-Au NPs). PSH dissociates Rh6G-Au NPs complexes and releases Rh6G molecules into the reaction media, which subsequently recovers fluorescence of Rh6G quenched by fluorescence energy transfer (FRET) between Rh6G and Au nanoparticles. The developed method was used for the determination of PSH in pharmaceutical formulations and serum samples. During the 24h interval following oral ingestion of 180 mg of PSH, serum concentrations ranging from ~0.1 to 0.8 μg mL^-1^ are expected [18]. The method can be appropriate for clinical pharmacokinetic evaluations after the consumption of the drug. The possible mechanism of interaction between the analyte and the nano sensoris studied and discussed. The proposed method can be used independently or in conjunction with various chromatographic or CE methods as their detection system. The method was optimized by the response-surface method (RSM), which is a powerful multivariable analysis tool that can provide us with a global optimum condition of the system and can reveal any possible interactions between the investigated experimental factors [19–21].

## 2. Materials and methods

### 2.1. Materials

All chemicals were purchased from Merck KGaA (Darmstadt, Germany), unless stated otherwise. PSH was obtained from Sigma-Aldrich Chemie GmbH (Taufkirchen, Germany). Deionized doubly distilled water was obtained from Kasra Company (Tabriz, Iran) and was used throughout. Fifty mmol L^-1^ of hydrogen tetrachloroaurate stock solution was prepared in a 50 mL volumetric flask by dissolving 1.00 g of the salt in water and making the solution to volume. For preparing buffer solutions, appropriate amounts of relevant salts were dissolved in water and subsequently, the pH of the solutions was adjusted by concentrated sodium hydroxide and hydrogen chloride solutions; the solutions were then made to the appropriate volume by adding water. The stock solution of PSH was prepared daily and kept refrigerated.

### 2.2. Apparatus

An RF-5301PC spectrofluorometer (Shimadzu Corp., Kyoto, Japan) with a quartz cell (1 cm ×1 cm) was used for fluorimetric measurements. The wavelength of excitation was set at 520 nm. A UV-1800 Shimadzu spectrophotometer was used for obtaining UV-visible absorption spectra. Reaction times were measured by a stopwatch. A Philips transmission electron microscope (working potential set at 40 kV) was used for taking transmission electron microscopic (TEM) images. A Hanna model 211 digital pH-meter (Hanna Instruments, Rhode Island, USA ) was used for pH control of the solutions.

### 2.3. Synthesis of Au NPs and Rh6G-modified Au NPs

Spherical Au NPs were synthesized by a method previously reported in the literature [22]. The synthesized particles were then modified by Rh6G molecules. Briefly, 500μL of 2% (w/v) solution of hydrogen tetrachloroaurate were diluted with distilled water to 94.5 mL and the diluted solution was heated under reflux. As the solution began boiling, 5.00 mL of 1.1% (w/v) trisodium citrate solution were dribbled into the boiling solution, which was still under reflux and was being stirred. In this process, citrate acted as both a reducing and a stabilizing agent. Upon the color of the solution changing to red, heating was stopped and the solution was stirred for a further 2 min. The solution was then cooled and transferred into a 100 mL volumetric flask (its volume was adjusted to 100 mL by adding distilled water, if necessary). The synthesized Au NPs were stored in the refrigerator. The surface of synthesized Au NPs was modified by Rh6G to yield Rh6G-Au NPs. With this aim, 1.00 mL of the synthesized Au NPs was poured into a test tube containing 500 μL of Rh6G solution and the mixture was mixed for a few seconds.

### 2.4. Sample preparation

Serum samples were prepared according to a reported procedure [19]. In brief, a 1.00 mL aliquot of serum was transferred into a centrifuge vessel and 2.00 mL of 0.1 M Ba(OH)_2_ and 2.00 mL of 0.1 M ZnSO_4_ were added to precipitate the proteins. Then, the solution was centrifuged at 4000 rpm for 15 min. The supernatant solution was used as the sample solution. As for the cough-cold syrup, a 100 μL portion of the syrup was transferred into a 100 mL volumetric flask and it was filled to the mark by distilled water. This was used as the sample solution.

### 2.5. General procedure

To a 5 mL volumetric flask, 2.50 mL of Rh6G-Au NPs solution and 1.00 mL (serum) or 500 μL (syrup) of the sample solution were added. The solution was brought to volume by tris buffer (9.0 mmolL^-1^, pH = 7.05) and mixed gently. The obtained solution was kept still for 4 min to equilibrate at room temperature. An identical solution was prepared as the blank in exactly the same manner with the exception of the sample solution, which was replaced by distilled water. Fluorescence intensity of the sample ( *F* ) and blank ( *F_B_* ) was measured at 560 nm (λ_*exc*_ = 520 nm) and the analytical signal was defined as their difference (Δ *F* = *F-F_B_* ).

## 3. Results and discussion

### 3.1. Effect of PSH on Rh6G-Au NPs complex

**Figure 1:**
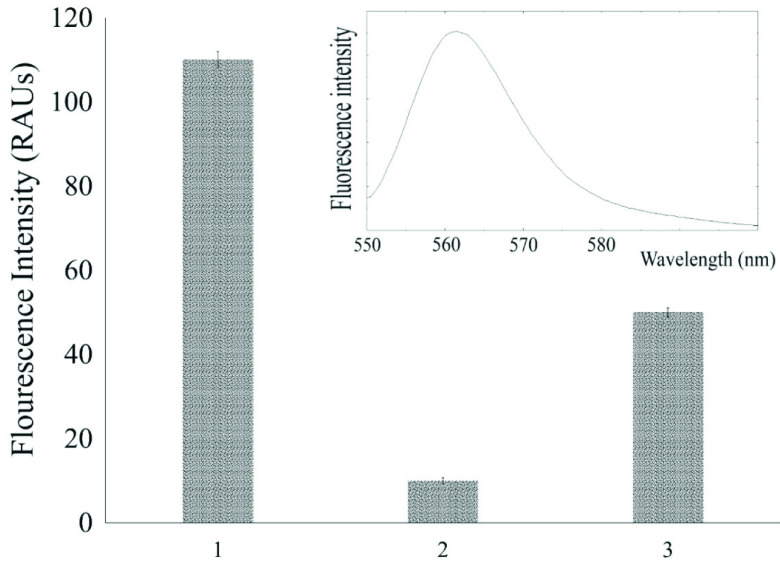
The intensity of fluorescence of Rh6G (5 ×10^-5^ mol L^-1^) (1); Rh6G-modified Au NPs (1.5 mL of 5 ×10^-5^ mol L^-1^ Rh6G mixed with 1000 μL Au NPs solution) (2);and Rh6G-modified Au NPs after addition of PSH (500 μL of 200 ng mL^-1^ PSH added to the solution in 2) (3). Inset: fluorescence spectrum of Rh6G.

The Rh6G with Au NPs complex was used as a chemical nanosensor for sensing and measuring PSH. Rhodamine 6G is a well-known fluorescent dye that is adsorbed noncovalently onto Au NPs. The interaction of Au NPs and Rh6G is governed by both the π-π and electrostatic interactions between the negative citrate moieties on the surface of the nanoparticles and the positively charged functional groups of the Rh6G dye. When Rh6G is closely located in the neighborhood of Au NPs, its fluorescence is effectively quenched through FRET and electron transfer (ET) processes [23]. The quenched fluorescence can be regained by detaching Rh6G from Au NPs, which in turn causes energy transfer processes (FRET and ET) to cease. It was observed that by adding PSH to Rh6G-Au NPs solution, the fluorescence of Rh6G molecules was recovered (Figure 1).

**Figure 2:**
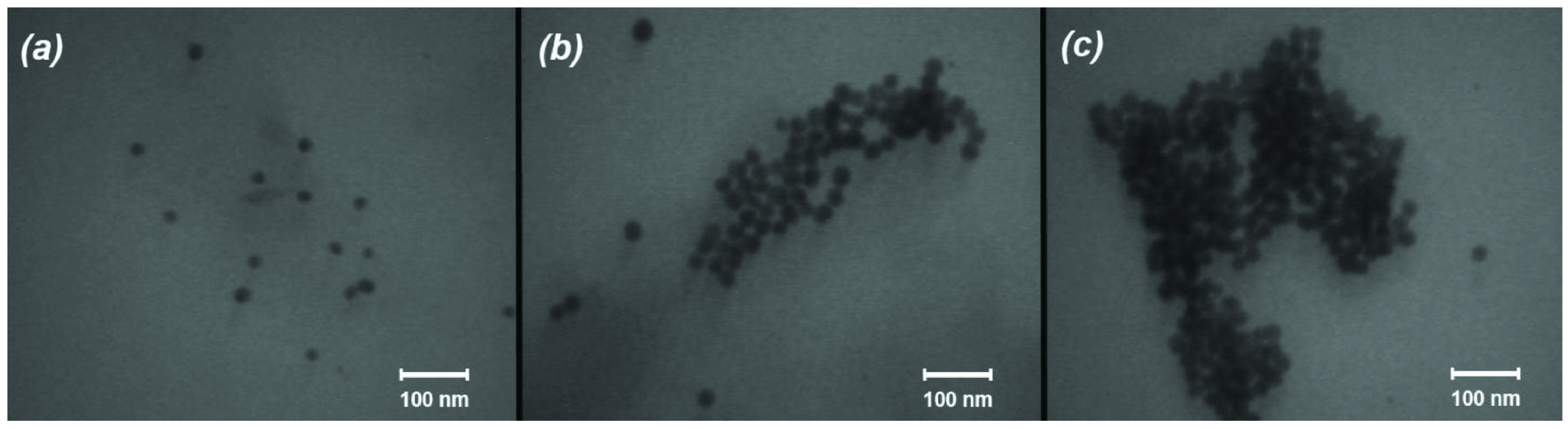
TEM images of Au NPs (a), Rh6G-modified Au NPs (b), and Rh6G-modified Au NPs after the addition of PSH.

The proposed mechanism was confirmed by TEM images (Figure 2). Figure 2a depicts the TEM image of pure, as-synthesized Au NPs before being treated by Rh6G. The synthesized spherical Au NPs had an approximate average diameter of 16 nm. Figure 2b depicts a TEM image of Rh6G-modified Au NPs. It shows the formation of some aggregates emerging from adsorption of Rh6G molecules on the surface of NPs, which bring and keep Au NPs close to one another. Figure 2c shows that by adding PSH to the latter solution results in the formation of bulkier aggregates that can be attributed to the replacement of Rh6G by PSH.

**Figure 3:**
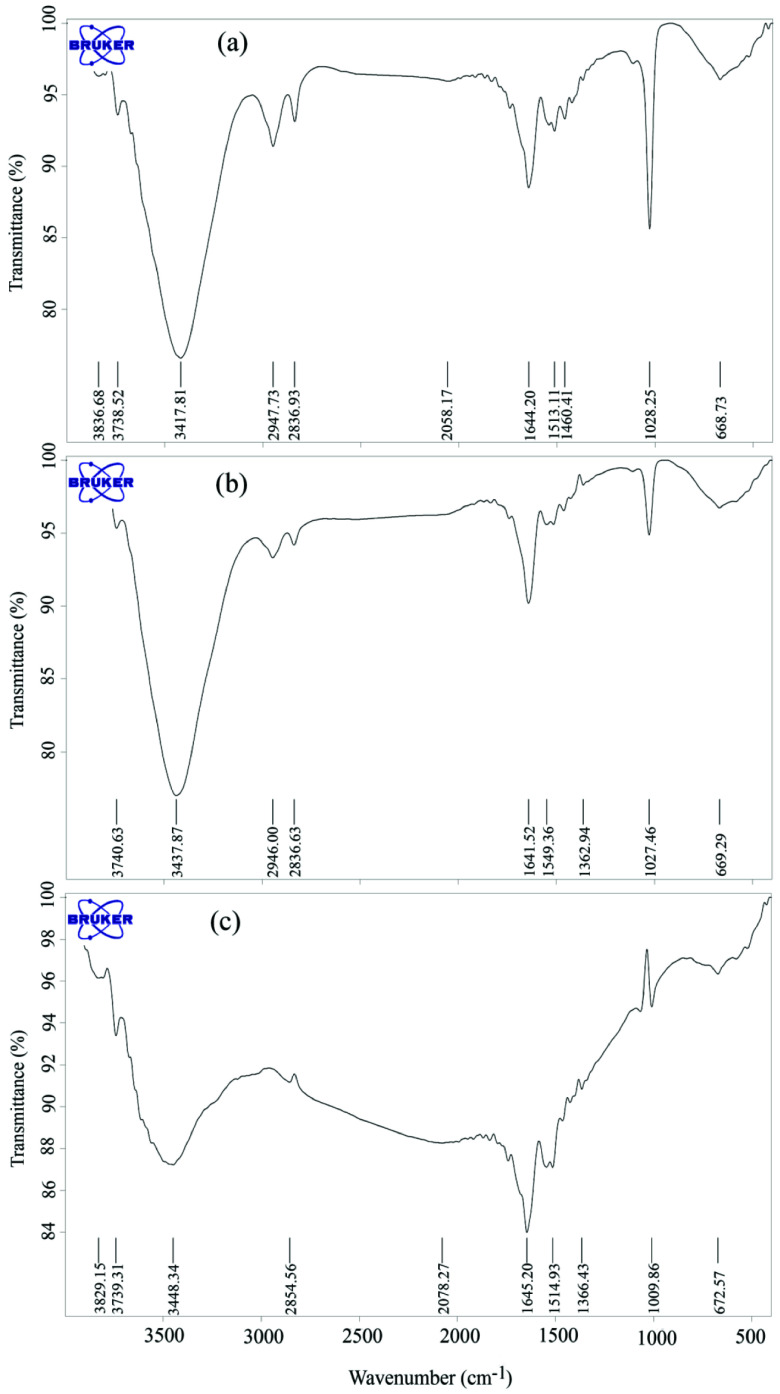
FT-IR spectrum of (a) gold nanoparticles (Au NPs); (b) Rh6G-Au NPs complex (b); and (c) RhB-Au NPs after adding PSH.

The above justifications were also confirmed by Fourier transform infrared (FT-IR) spectra. Figure 3a shows the IR spectrum of the gold nanoparticles. The absorption bands at 2800–3000 nm can be related to the hydroxyl group of citrate anion; the strong absorption band at 1640 nm can be ascribed to the asymmetric stretching vibration of the carboxylic group, which corroborates the presence of citrate on the particles. Upon treating Au NPs with Rh6G (Figure 3b), the hydroxyl vibrational bands were attenuated, which shows the interaction of citrate moieties with Rh6Gs. Figure 3c depicts an FT-IR spectrum of Rh6G-Au NPs after adding PSH. In the third spectrum, the peaks corresponding to the hydroxyl group disappeared, indicating stronger interactions between citrate moieties and PSH, which in turn results in the replacement of Rh6G with PSH and formation of larger aggregates. All these explanations were also observable in the recorded changes in the maximum fluorescence intensity of sample solutions, an example of which can be seen in Figure 1.

**Figure 4:**
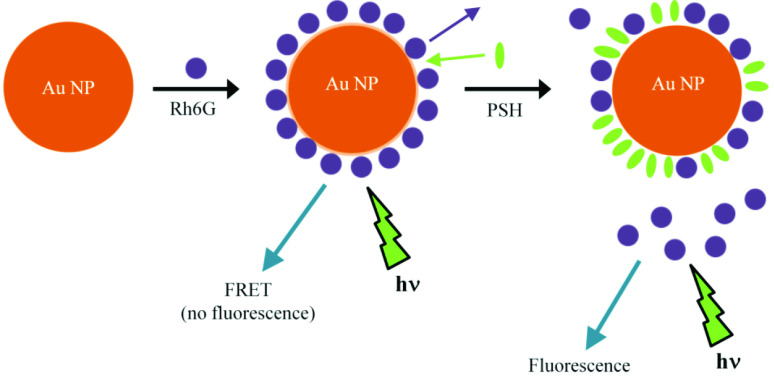
Schematic representation of the reaction used for analytical determination in the presence of Tris buffer (pH = 7.05).

To sum up, a schematic representation of the analytical reaction is shown in Figure 4.

### 3.2. Optimization of the reaction conditions

The response surface methodology is a clever combination of techniques that take root in mathematics and statistics used for solving various optimization problems. It helps researchers find the global optimum conditions of the system rather than any likely local optima [19–21]. The present study takes advantage of central composite design (CCD), a widely used version of RSM [19,20], for optimizing the analysis conditions in terms of achieving the largest possible *ΔF* values. Constructing a table of design and statistical analyses of the obtained results was done by Minitab® 15 software. Using CCD, the effect of three (n=3) experimental factors were investigated: pH of the buffer, the concentration of the buffer, and concentration of Au NPs. Tris buffer was chosen among 3 tested buffers (phosphate, acetate, and tris buffer). While time had no effect on *F_B_* , the time of the reaction between PSH and Rh6G-Au NPs had some influence on the measured *ΔF* . Fluorescence intensity measured at different time intervals for blank and PSH samples showed that 3 min was the minimum time to reach maximal fluorescence difference signals. Thus, fluorescence signals were measured 3 min after the reagents were mixed. The concentration of Rh6G was not included in the design because for any given amount of Au NPs the concentration of Rh6G should be tuned to a certain level. This “certain level” was determined in the following way: for any needed volume of Au NPs solution, the corresponding maximum amount of Rh6G was found for which the intensity of fluorescence of their mixture was not significantly larger than the background fluorescence signal plus 3σ of the background fluorescence signal (background signal was the fluorescence signal measured in the presence of buffer and Au NPs, without Rh6G). These investigations determined the amount of Rh6G that was needed to saturate Au NPs by organic dye with minimal free Rh6G in the solution. During optimization experiments, the concentration of PSH was fixed at 100 ng mL^-1^.

The CCD was composed of 2 *n* cubic points, 2 *n* axial points set at a distance of α = 2 from the center point, and *r* = 5 replicate determinations of the center point. The total number of design experiments was 19 (for n= 3). Prior to any mathematical analyses, the variables *X_i_* were transformed into coded ones, *x_i_* , according to equation (3) [19]:

(3)xi=(Xi-X0)δX

where *X_0_* is the value of *X_i_* at the center point and *δX* shows the absolute value of the difference between *X_i_* at any level with its adjacent level. The working range of the factors was chosen according to preliminary tests and experience. The influence of pH was studied at levels ≥ 5 because at lower values Au NPs are unstable and undergo aggregation. The coded levels of the studied variables, together with their actual amounts, are tabulated (Table 1). The 3-factor design table, along with an experimental signal used for modeling the system, can be seen in Table 2. The obtained data were analyzed by regression to calculate the coefficients (as) of the response model which was initially assumed by CCD to be a full second-order polynomial:

(4)R=a0+∑i=13aixi+∑i=33aiixi2+∑i=13∑j=2(j≠i)3aijxixj+E

where *R* designates the response, *a_0_* is the intercept, *x_i_* s designates the coded factors, *a_i_* s, *a_ii_* s, and *a_ij_* s designate the linear parameters, the quadratic parameters, and the interaction parameters, respectively. All these parameters were calculated by the least-squares method. The significance of each parameter was assessed on the basis of its corresponding P-value and after putting aside insignificant parameters (in this work, those pertaining to *x_13_* and *x_23_* ) at the 95% confidence level (i.e. P < 0.05), the least-squares matrix was reestablished for obtaining a new set of values of the parameters. The parameters were assessed again for their significance by taking advantage of the analysis of variance (ANOVA). The estimated values of the significant parameters, being retained from equation (2), were calculated by the least-squares method and are given in Table 3.

**Table 1:** Factor levels of CCD.

Coded levels	Variables and their actual levels [symbol used for the variable]		
	pH	Au NPs (μL)	Buffer concentration (mol L^-1^)
	[pH]	[Vol.]	[Conc.]
+2	11.5	1600	2.5 x 10^-2^
+1	10	1300	2.0 x 10^-2^
0	8.5	1000	1.5 x 10^-2^
-1	7	700	1.0 x 10^-2^
-2	5.5	400	0.5 x 10^-2^

**Table 2:** The 3-factor composite design matrix with experimental values and predicted response function values for each run.

Run order	pH	Au NPs (μL)	Buffer concentration (mol L^-1^)	Response (Δ F)	
Experimental	Predicted
1	0	0	0	301	302.838
2	-1	$+1$	-1	322	324.530
3	0	-2	0	144	147.595
4	$+2$	0	0	154	155.345
5	0	0	$+2$	160	151.595
6	0	0	0	306	302.838
7	-1	-1	$+1$	207	209.030
8	$+1$	$+1$	-1	236	227.780
9	0	0	0	303	302.838
10	$+1$	-1	$+1$	162	160.280
11	$+1$	-1	-1	259	255.780
12	-2	0	0	304	300.845
13	0	0	0	298	302.838
14	0	$+2$	0	145	139.595
15	0	0	-2	336	342.595
16	0	0	0	308	302.838
17	-1	-1	-1	307	304.530
18	-1	$+1$	$+1$	223	229.030
19	$+1$	$+1$	$+1$	120	132.280

**Table 3:** Regression and analysis of variance (ANOVA) of CCD.

Parameter	Parameter estimate	Standard deviation	P-value
a_0_	302.838	2.980	0.000
a_1_	-36.375	1.713	0.000
a_2_	-2.000	1.713	0.268
a_3_	-47.750	1.713	0.000
a_11_	-18.686	1.408	0.000
a_22_	-39.811	1.408	0.000
a_33_	-13.936	1.408	0.000
a_12_	-12.000	2.422	0.000

The optimum point was predicted by taking advantage of the Minitab® response optimizing function. The optimum coordinates and the response arising from using them are given in Table 4.

**Table 4:** Optimum levels of factors and the optimum response.

Variable	Coded level	Decoded level	Predicted optimum response [Experimental optimum response]
pH	–1.030	7.05	362
Au NPs solution volume (μL)	0.141	1042	[353]
Buffer concentration (mol L^-1^)	–1.711	0.009	

**Figure 5:**
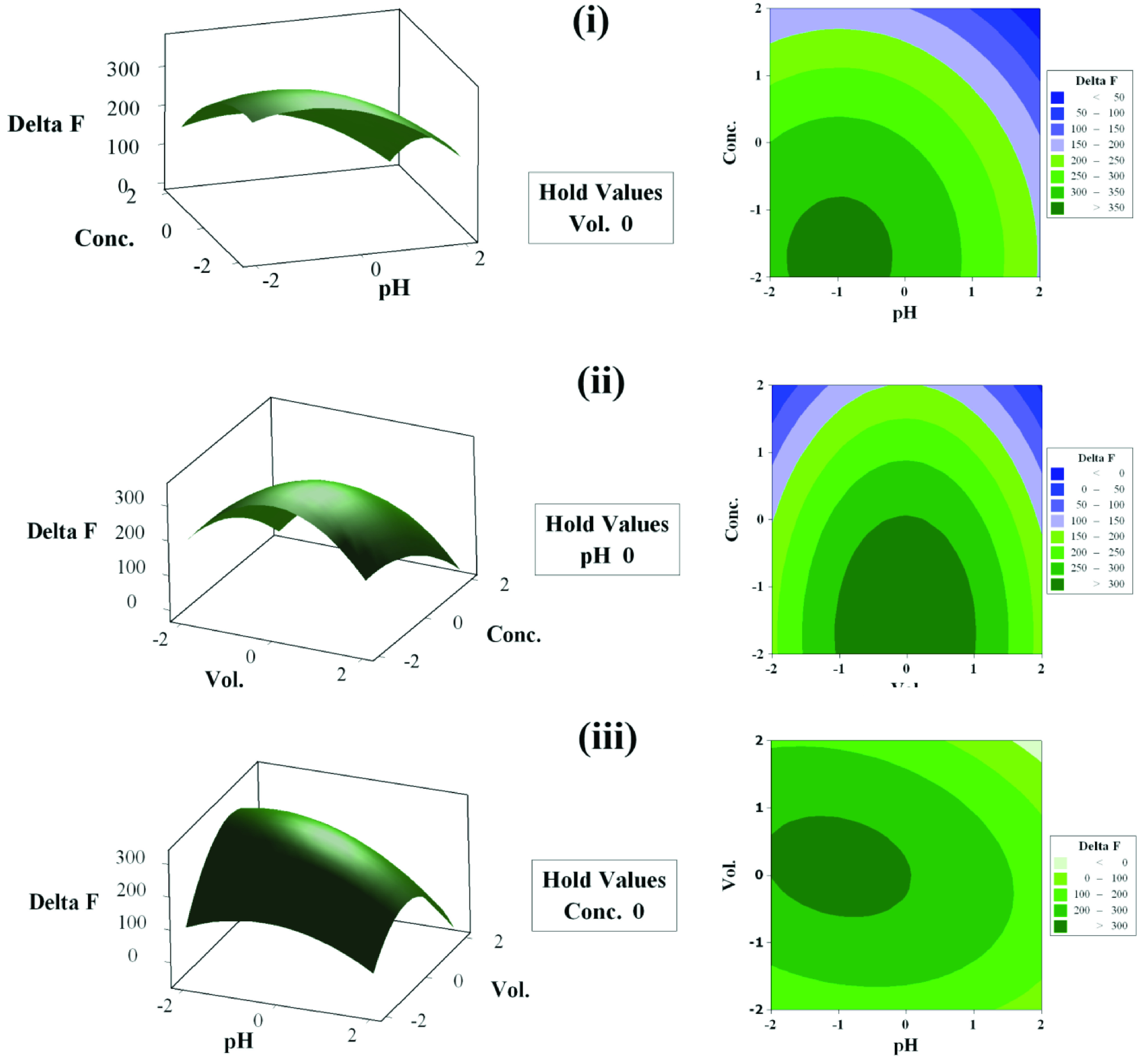
The contour plots and surface plots of ΔF as a function of pH and concentration of Tris buffer (i); concentration of the buffer and volume of Au NPs solution (ii); and pH and volume of AuNPs solution (iii). At each part of the figure, the other two factors were kept constant at their center level.

Figure 5 depicts contour plots along with surface plots of the pertinent 5-level RSM experiments conducted for optimizing the conditions of the analysis. The plots were drawn with a factor of the constant level being set at its middle level. The contour lines, which present cocenter ellipses, show that there is no significant interaction among the factors. They also show the nonlinearity of the effect of the variables on the response.

**Figure 6:**
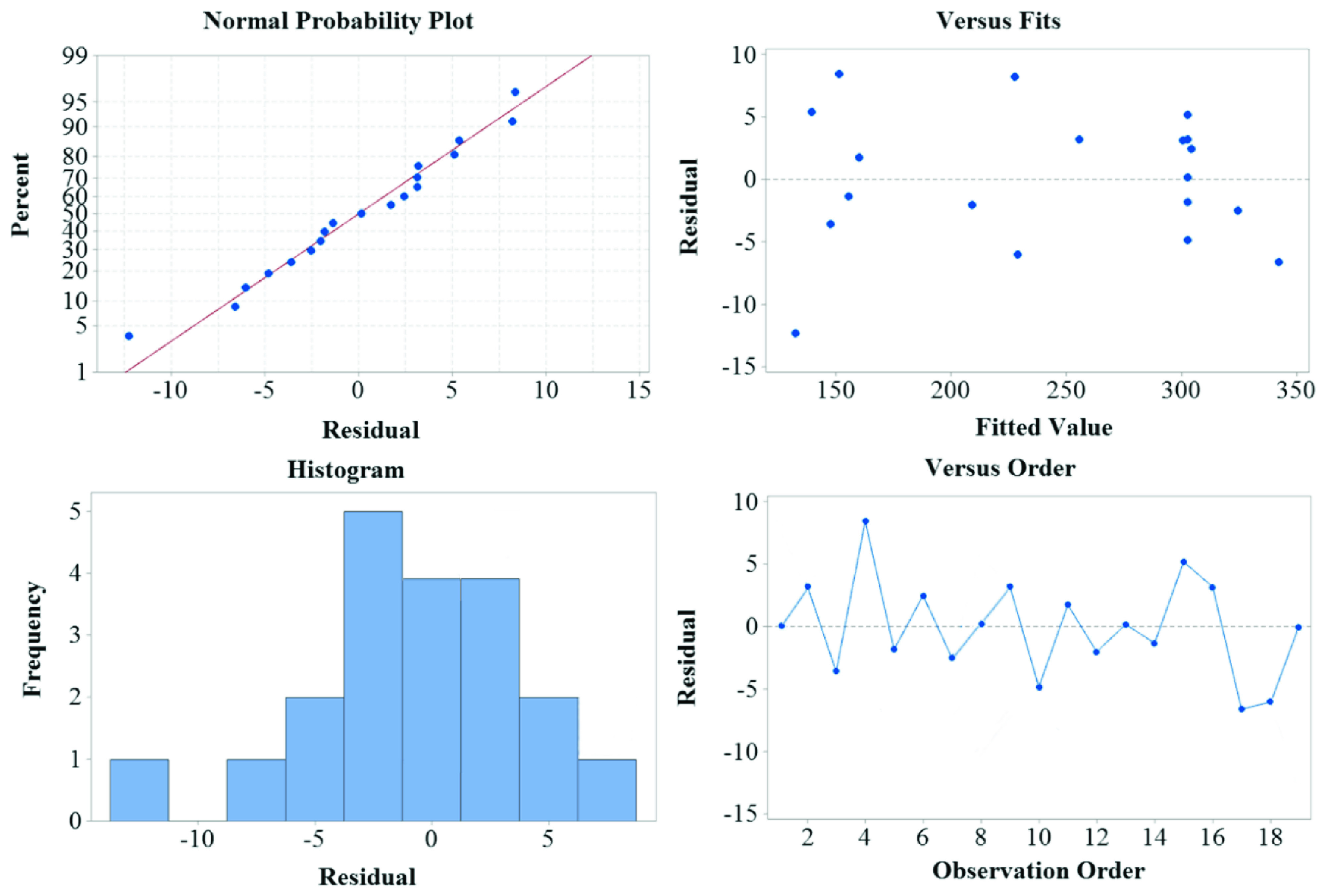
Residual plots for analytical fluorescence signals.

The adequacy of the models was evaluated using the residuals (the difference between the observed response value and the predicted response) (Figure 6). Residuals are considered as part of variations not explained by the fitted model and their occurrence is expected to possess a normal distribution. The normal probability plot is a suitable graphical tool to judge if the residuals are normally distributed. The observed residuals were plotted versus the expected values given by a normal distribution. The trends in Figure 6 show reasonably well-behaved residuals. Based on these plots, the residuals appear to be randomly scattered, which can confirm the adequacy of the model.

## 4. Analytical applications

### 4.1. Figures of merit

**Figure 7:**
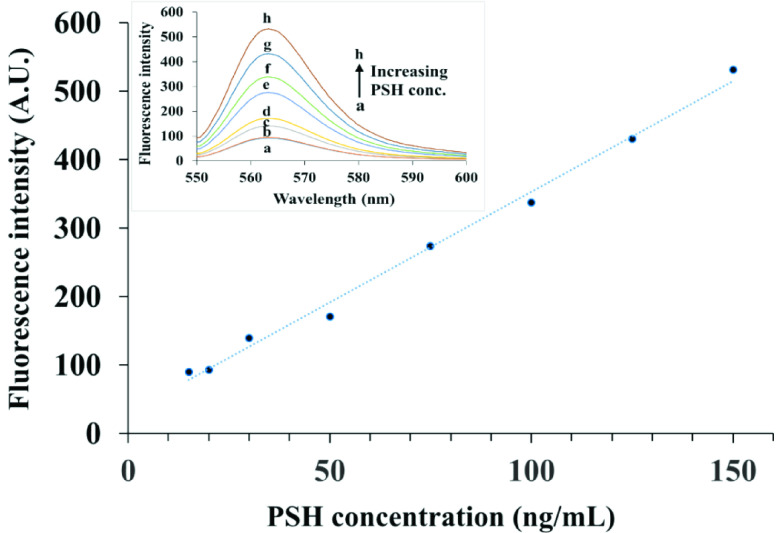
Calibration curve for determination of PSH in the linear range of the method. Inset: Fluorescence spectra related to the calibration points.

The observed fluorescence intensity recovery was found to be proportional to the concentration of PSH and was used as a basis for the determination of PSH. The analytical signal was linear over the range of 15–150 ng mL^-1^. The calibration data can be seen in Figure 7. The mathematical equation of the regressed line was *ΔF* = 3.234C+ 30.151, where *ΔF* is the analytical signal andC is PSH concentration expressed in ng mL^-1^. The squared correlation coefficient of the calibration graph was equal to r^2^ =0.9933 and the detection limit was equal to LOD = 10 ng mL^-1^. The detection limit (LOD) was calculated according to the IUPAC definition (three times the standard deviation of the blank signal divided by the slope of the calibration line) [24]. Percent relative standard deviation of the method for five repeated determinations of 50 ng mL^-1^ PSH was equal to 3.74%, which shows good repeatability of the method.

Table 5 compares the analytical figures of merit of the proposed method with those of some previously published methods. The proposed method offers analytical characteristics that are better than its fluorimetric predecessor [17]. As compared to some methods [1], it does not need any expensive equipment or laborious pretreatment step, and compared to others [10] it does not need sophisticated mathematical calculations. The range of applicability of the proposed method and its LOD make it a good candidate for pharmacokinetic evaluations related to PSH.

**Table 5:** Comparison of analytical parameters of the proposed method with some methods reported in the literature.

Method	Linear range (ng mL^-1^)	LOD (ng mL^-1^)	Reference
Liquid chromatography	1.50 x 10^5^ - 6.00 x 10^5^	7.50 x 10^2^	[1]
Spectrophotometry	1.00 x 10^5^ - 1.10 x 10^6^	1.291 x 10^4^	[11]
Spectrophotometry	5 x 10^3^ - 3.0 x 10^4^	2 x 10^3^	[10]
Voltammetry	up to 1400 ^a^	4.17	[15]
Spectrofluorimetry	5.00 x 10^2^- 5.000.0 x 10^3^	112	[17]
Spectrofluorimetry	15-150	10	The proposed method

^a^ The lower limit was not reported.

### 4.2. Interferences

The effect of some common chemical species on the analytical signal was studied to assess the analytical applicability of the method. Samples containing 60 ng mL^-1^ of PSH and increasing amounts of the investigated chemical species were analyzed by the proposed method. The limit of toleration for each species was defined as that resulting in a relative error of no more than 5%. The results are given in Table 6. The data show that the proposed method has good selectivity and can be applied in real matrices of interest. The cough-cold syrups usually contain about 0.4 mg mL^-1^ of sodium saccharin. As can be seen, the method tolerates this species well.

**Table 6:** Limits of toleration (concentration ratios for some chemical species). Analyte concentration: 60 ng mL^-1^ (or .3.0 ×10^-7^ mol L^-1^).

Species	Tolerable concentration ratio^a^ *(C_species_/C_PSH_)*
Na^+^, K^+^, F^-^, Cl^-^, NO^-^_3_, SO^2-^_4_	3000
Sodium saccharin, uric acid, glucose, sucrose, lactose, fructose, galactose, ascorbic acid, sorbitol, oxalate	1500
Ba^2+^, Mg^2+^, Ca^2+^, Cu^2+^, Co^2+^, Ni^2+^, Zn^2+^, Cd^2+^, Mn^2+^, PO^3-^_4_, Fe^3+^, Al^3+^, NH^+^_4_, Br^-^	1000

^a^ Concentration ratios for molecular species were based on ng mL^-1^ and for ionic species on molar concentrations.

### 4.3. Applications

The proposed method was applied to determine the amount of PSH in cough-cold syrup and human blood serum samples. The results obtained for the pharmaceutical formulations were in good agreement with the results obtained using an official method [25]. The validity of the proposed method was also assessed by recovery tests done on spiked samples. The t-test was used to confirm the accuracy of the results and their equivalence to accepted or known values. A summary of the results is given in Table 7. The null hypotheses for t-tests were set as zero difference between the added and recovered amount (for spiking tests), and zero difference between the concentration measured by the proposed method and that measured by the official method (for samples measured by both methods).

**Table 7:** Results for the analysis of PSH in serum and pharmaceutical formulations.

Sample	Added^a,b^	Found^a,b^ by (±S.D.)		Recovery (%)	t-statistic^c^
					
Cough-cold syrup^d^ (Caspian Tamin^TM^)	0 1 2	5.91 (±0.19) 6.90 (±0.22) 7.95 (±0.20)	5.93 (±0.17) 6.92 (±0.15) 7.93 (±0.19)	- 99 102	0.16 0.15^e^, 0.09^f^ 0.14^e^, 0.4^f^
Serum	0 200 400 700	Not detected 202 (±12) 415 (±17) 687 (±27)	- - - -	- 101 104 98	- 0.33 1.76 1.63

^a^In mg mL^-1^in cough-cold syrup and in ng mL^-1^ in serum. ^b^Mean of four repeated determinations. ^c^Critical value of t for three and six degrees of freedom at a 95% probability level is equal to 2.78 and 2.45, respectively. ^d^Nominal content: 6 mg mL^-1^. ^e^t-value calculated on the basis of official method analysis. ^f^ t-value calculated on the basis of spiking/recovery tests.

## 5. Conclusion

An inexpensive, simple, sensitive, and rapid method was developed to determine the amount of PSH in pharmaceutical and serum samples. To the best of the authors’ knowledge, this is the second fluorimetric method applied to determine the quantity of PSH, but it enjoys a linear dynamic range and LOD lying at much lower concentration levels. This, combined with the good selectivity of the method, turns the method into a promising analytical tool for pharmacokinetic evaluations after consumption of the drug. Also, owing to the low LOD of the method, it can be used independently or as a detection aid to be coupled with liquid chromatographic or electrophoretic systems. The experimental factors were optimized using a powerful RSM methodology, namely CCD methodology, which allowed global optimization rather than local optimization of the system.
